# Translation and Validation of the Korean Version of the Revised Health Care System Distrust Scale (HCSD-K) in Korean American Women

**DOI:** 10.3390/ijerph15091964

**Published:** 2018-09-08

**Authors:** Hye Chong Hong, Ari Min

**Affiliations:** Mo-Im Kim Nursing Research Institute, College of Nursing, Yonsei University, Seoul 03722, Korea; julie9278@gmail.com

**Keywords:** Health Care System Distrust scale, reliability, validity, Korean American, women

## Abstract

Background: Trust in the healthcare system is a major contributor for racial disparities in health and health care. We aimed to formally translate and cross-culturally adapt the Korean version of the Revised Health Care System Distrust (HCSD-K) scale with a sample of Korean American women and examine the psychometric properties of the HCSD-K scale. Methods: Ten Korean American women participated in the cognitive interview. A self-administered questionnaire was completed by 196 Korean American women aged 50–74 years. Instrument adaptation was performed using committee-based translation and cognitive interviewing. Construct validity, convergent validity, and internal consistency were examined to evaluate the psychometric properties of the HCSD-K scale. Results: The translated instrument was found to be semantically sound. A confirmatory factor analysis revealed a two-factor structure with an excellent fit. Convergent validity was supported by correlations between the HCSD-K scale and both the Perceived Discrimination in Health Care and Trust in Physician scales. Cronbach’s alpha for the total HCSD-K was 0.83. Conclusion: The nine-item HCSD-K scale demonstrated satisfactory reliability and validity. It is an appropriate instrument for measuring healthcare system distrust in Korean American women. Further study is needed to confirm the study results in a gender-mixed Korean population.

## 1. Introduction

Trust is defined as “the willingness of a party to be vulnerable to the actions of another party based on the expectation that the other will perform a particular action important to the trustor, irrespective of the ability to monitor or control that other party” [1]. In health care, trust has been distinguished or categorized by the object of trust, such as trust in the physician and/or trust in the healthcare system, which is characterized by attitudes toward collective healthcare-related organizations [2,3,4,5]. Trust in the healthcare system is a major contributor for racial disparities in health and health care and has become a priority for health research and healthcare delivery systems [6,7,8]. There is a growing body of literature indicating that trust in health care is associated with healthcare utilization in minorities. For example, trust in the healthcare system was found to be a strong predictor for breast and cervical cancer screening, even when socioeconomic variables were controlled [8,9,10]. Most studies related to trust in the healthcare system and healthcare utilization were conducted with African Americans and Hispanics. 

Korean Americans (KAs), including individuals born both in Korea and the United States, are the fifth-largest Asian American ethnic group in the United States [11,12]. The number of KAs has increased more than 60%, from 1 million to 1.8 million between 2000 and 2010, and as such KAs represent one of the fastest growing Asian groups in the United States [13]. KAs continue to have lower rates for cancer screening and healthcare utilization [14,15,16]. Several interventions incorporating healthcare access, health beliefs, and cultural factors have been developed and delivered to KAs, but their cancer screening and healthcare utilization rates still remain very low [12,17]. Trust has been identified as a critical factor that influences health service utilization, health status, and patient satisfaction in other populations [18,19,20,21]. Among KAs, trust in the healthcare system is an understudied area and may be able to partially explain the low rate of healthcare utilization. However, trust in health care had not been measured in the KA population as no valid instrument was available.

The Revised Health Care System Distrust (HCSD) scale was developed by Shea et al. [22] to assess general beliefs about the healthcare system in diverse racial and ethnic groups. Unlike previous scales measuring healthcare trust with a single dimension, the Revised HCSD scale was developed using factor analysis, which yielded competence distrust (“perception that the entity is (not) capable of doing what is needed” [22]) and values distrust (“perception that the entity (does not) wants to do what is needed” [22]). Yang et al. [10] also confirmed the two factors using factor analysis. However, items loaded on each factor differed slightly between the studies of Shea at al. [22] and Yang et al. [10]. 

The purpose of the present study was to formally translate and cross-culturally adapt the Korean version of the Revised HCSD (HCSD-K) scale with a sample of KA women and to examine the psychometric properties of the HCSD-K scale. Three specific aims were established to: (1) translate the Revised HCSD scale into Korean, (2) examine the construct validity of the HCSD-K scale, (3) and examine the reliability of the HCSD-K scale. The goal of developing the HCSD-K scale was to provide a simple and easy-to-use reliable and valid instrument for researchers and healthcare providers to assess healthcare system distrust levels in Korean Americans.

## 2. Materials and Methods

### 2.1. Participants

This study was a part of a cross-sectional study examining the effects of perceived discrimination and trust regarding breast cancer screening among KA women [23]. The target sample was composed of KA women living in Chicago and the greater metropolitan areas. A convenience sampling was used. We contacted major Korean churches in Chicago and the surrounding metropolitan areas because (1) more than 70% of the KA population attend Korean churches, and (2) KAs tend to seek health care information from church and church members. [24,25] We then asked permission from the key personnel (pastors or gatekeepers) in the four Korean churches to recruit participants before and after worship services on Sundays. With permission, a table was set up with study flyers. Once women showed interest in the study, they were screened for eligibility. A total of 196 KA women aged between 50 to 74 participated in the study. The study design and sampling methods are explained in detail elsewhere [23]. 

Data collection began after obtaining approval from the Institutional Review Board (IRB) at a university (Research Protocol # 2016-0528). Informed consent was waived as minimal personal information and minimal risks were presented in this study. Women who were willing to participate were asked to read the information letter. Before agreeing to participate in the study, we carefully explained the study purpose, potential benefits, and time commitment of the study to each potential participant. The participants were asked to answer questionnaires based on their experiences with healthcare system and healthcare providers in general. Surveys took approximately 15 to 20 min to complete.

### 2.2. Measures

#### 2.2.1. Healthcare System Distrust

From the diverse racial/ethnic focus group interview and discussion, a Revised HCSD scale consisting of nine items was developed: “The Health Care System does its best to make patients’ health better” (item 1), “The Health Care System covers up its mistakes” (item 2), “Patients receive high quality medical care from the Health Care System” (item 3), “The Health Care System makes too many mistakes” (item 4), “The Health Care System puts making money above patients’ needs” (item 5), “The Health Care System gives excellent medical care” (item 6), “Patients get the same medical treatment from the Health Care System, no matter what the patient’s race or ethnicity” (item 7), “The Health Care System lies to make money (item 8), and “The Health Care System experiments on patients without them knowing” (item 9) [22]. The nine items are rated on a five-point Likert-type scale (strongly disagree, disagree, neither agree nor disagree, agree, or strongly agree), with a possible distrust score ranging between 9 and 45. Items 1, 3, 6, and 7 are reverse coded, so a higher score denotes higher distrust (or less trust) in the healthcare system. The scale consists of two subscales: (1) competence distrust (4 items; Cronbach’s alpha = 0.77), and (2) values distrust (5 items; Cronbach’s alpha = 0.73). In the Revised HCSD scale by Shea et al. [22], the authors categorized items 1, 3, 4, and 6 into competence distrust, although item 4 loaded on values distrust. The authors stated that item 4 was conceptually closer to competence distrust [22]. Moreover, Shea et al. [22] categorized items 2, 5, 7, 8, and 9 into values distrust, even though item 7 loaded on competence distrust, since these items were conceptually part of values distrust. The factor analysis (varimax rotation) performed by Yang et al. [10] showed two factors: items 1, 3, 6, and 7 loaded on competence distrust and items 2, 4, 5, 8, and 9 loaded on values distrust. 

In previous studies, both the reliability and validity of the Revised HCSD scale were found to be similar for African Americans and whites [10,22]. Values and competence distrust were negatively associated with the physician trust subscale of the Primary Care Assessment Survey (*r* = −0.30, *r* = −0.33), a global item assessing trust in the healthcare system *(r* = −0.42, *r* = −0.55), and a global item assessing general social trust from the General Social Survey (*r* = −0.35, *r* = −0.27), thus supporting construct validity [22]. This scale had not been used with KAs. 

#### 2.2.2. Perceived Discrimination in Health Care

Perceived discrimination in health care was measured by a modified version of Williams’ Everyday Discrimination Scale [26,27]. The Perceived Discrimination in Health Care scale comprises seven items that are scored on a five-point Likert-type scale (never, rarely, sometimes, most of the time, or always). Total scores range from 7 to 35, and a higher score indicates greater perceived discrimination. Cronbach’s alpha was 0.89 for African Americans, 0.60 for Latinas, and 0.94 for American Indians. Factor analysis confirmed one factor [28,29,30]. In KAs, Cronbach’s alpha was 0.90 for the nine-item version of the Everyday Discrimination Scale, and construct validity was supported by a significant relationship between perceived discrimination and depression (*r* = 0.38, *p* < 0.001) in a study with 304 Korean immigrants who resided in New York City [31]. In this study, Cronbach’s alpha was 0.88.

#### 2.2.3. Trust in Physician 

The Trust in Physician scale was developed to assess the level of interpersonal trust in the patient-physician relationship [32]. The Trust in Physician scale consists of 11 items and each item is rated on a five-point Likert-type scale that ranges from “strongly disagree” to “strongly agree”, producing a total score from 11 to 55. Participants with a higher score have higher trust in healthcare providers. Cronbach’s alpha for the original scale was 0.90, and 0.84 for the Chinese version of Trust in Physician scale [33]. Construct validity was confirmed by a significant correlation between the Trust in Physician scale and satisfaction with physicians in two studies (*r* = 0.62, *p* < 0.001; *r* = 0.73, *p* < 0.001) and between the Trust in Physician scale and desire for clinician’s control (*r* = 0.48, *p <* 0.001) [32,34]. In this study, Cronbach’s alpha was 0.82.

### 2.3. Translation Process

#### 2.3.1. Step 1: Committee-Based Translation

The committee-based translation method is an approach to decrease the introduction of cultural bias inherent in the native language by collaborative and consensus translation efforts [35,36]. In contrast to the Brislin forward-backward translation approach, committee-based translation maximizes the retention of concept meaning that may be altered during forward-backward translation. Three bilingual Korean PhD students, who had research experience and familiarity with both Korean and Western culture, were asked to translate the scale. Each translator independently translated the scale and several meetings were held to discuss, resolve, and adjudicate the translation.

#### 2.3.2. Step 2: Expert Review

An expert who had experience in translating instruments and who was familiar with both Korean and Western culture was invited to evaluate the translated version. This process enhanced the conceptual equivalence across cultures and thus added content validity. 

#### 2.3.3. Step 3: Cognitive Interviews

The translation of the scale was pre-tested using cognitive interviews with 10 KA women aged between 50 and 74 years old. Cognitive interviews were used during the pre-testing phase of the questionnaire to detect items and words that were not understood by the participants as intended by the researchers [37]. Verbal probing techniques were used during the cognitive interview [38,39]. Each participant was asked the survey questions. If there were differences in the interpretation of the questions, the translation was reviewed for modification by the translators.

#### 2.3.4. Step 4: Review of Translated Scale

Cognitive interviews ensured that KA women clearly understood the translation. The final scale was taken back to the expert for final confirmation that the translated scale was clear and conceptually equivalent.

### 2.4. Statistical Analysis

#### 2.4.1. Descriptive Statistics

All data analyses were conducted using STATA version 13.0 (StataCorp LP., College Station, TX, USA). There were no missing data because we were available to answer any questions and to check for missing data during the survey collection. Descriptive statistics, such as means, standard deviation, frequencies, and percentages, were used to describe the participants. 

#### 2.4.2. Construct Validity

A confirmatory factor analysis (CFA) was performed to test whether the two factors of the original Revised HCSD scale [22] could sufficiently describe the data of the Korean version (HCSD-K). For the HCSD-K scale, the two-factor model proposed by Shea et al. [22] was estimated first. Then, this model was compared to the modified two-factor model proposed by Yang et al. [10]. Barlett’s test of sphericity and the Kaiser–Meyer–Olkin measure of sampling adequacy were performed to evaluate the appropriateness of the factor model and sample size for the factor analysis. The result of the Chi-squared of Barlett’s test of sphericity with statistical significance at the 0.05 level indicated that a factor analysis of the data may be useful. Kaiser–Meyer–Olkin values between 0.8 and 1 indicate adequate sampling; values less than 0.5 indicate inadequate sampling and that remedial action should be taken [40,41]. Quality of model fit was assessed using the following fit indices and respective reference values: a root mean square error of approximation (RMSEA) below 0.06, a standardized root mean residual (SRMR) below 0.08, and a comparative fit index (CFI) and Tucker–Lewis index (TLI) larger than 0.95 are interpreted as denoting a good fit, whereas RMSEA ≤ 0.08 and CFI/TLI ≥ 0.90 are often considered as indicating acceptable fit [42].

Discriminant validity was assessed by comparing the average variance extracted (AVE) value of each latent construct with the shared variance (squared correlation) between constructs. This AVE value is the average amount of variance in observed items that a latent construct is able to explain. Discriminant validity is supported when the AVE of a latent construct is greater than its squared correlation with any other latent construct in the model [43]. 

Convergent validity was examined by assessing the scale using Pearson’s correlation coefficients. Convergent validity was calculated by correlating the scores obtained from the HCSD-K, Perceived Discrimination in Health Care, and Trust in Physician scales. We hypothesized that the HCSD-K and Perceived Discrimination in Health Care scales measure a similar construct; thus, their scores should be positively correlated. We also hypothesized that the HCSD-K and Trust in Physician scales measure a theoretically opposite construct; therefore, their scores should be negatively correlated.

#### 2.4.3. Reliability

Internal consistency was assessed using Cronbach’s alpha coefficients. A commonly accepted rule for describing internal consistency using Cronbach’s alpha values is as follows: alpha ≥ 0.9 is excellent, 0.8 ≤ alpha < 0.9 is good; 0.7 ≤ alpha < 0.8 is acceptable; and alpha < 0.5 is unacceptable [44,45]. Item-total scale correlation analyses were also completed to ensure the internal consistency within the scale. Usually, item-total correlation coefficients > 0.30 are regarded as acceptable [46]. 

## 3. Results

### 3.1. Participants

A convenience sample of 196 KA women aged between 50 and 74 years old completed the questionnaire (Table 1). Their mean age was 63 ± 6.78 years. More than 75% of KA women had lived in the USA for more than 20 years, with a mean length of stay of 29 ± 11.32 years (range: 2–54 years), and most were naturalized citizens (80%). Seventy-five percent of women were married and 59% were educated at the college level or higher. About one-third of the women reported household earnings above US$40,000 and 24% earned less than US$25,000 per household. Forty-four percent of women worked either full-time or part-time. The majority of KA women (85%) had a regular doctor or a regular place they could go for health care, and about 88% responded that their doctors were Korean. Most of the KA women (92%) responded that they were able to find a Korean doctor if they wanted, and about 80% of the women reported that their insurance allowed them to choose the doctor they preferred.

### 3.2. Translation, Cognitive Interviewing and Adaptation

Based on the committee-based translation method, a couple of adjustments were made to the HCSD-K scale. “Healthcare system” and “medical care” were translated differently between translators, therefore a consensus was reached after several discussions and confirmation from the expert. Cognitive interviews were conducted with 10 KA women and there were slight differences in the definition of healthcare system given by three women, so the questionnaire was taken back to the translators and expert to make adjustments. The final version of the questionnaire included a definition of healthcare system in the instructions to reduce confusion among the participants.

### 3.3. Construct Validity

**Figure 1:**
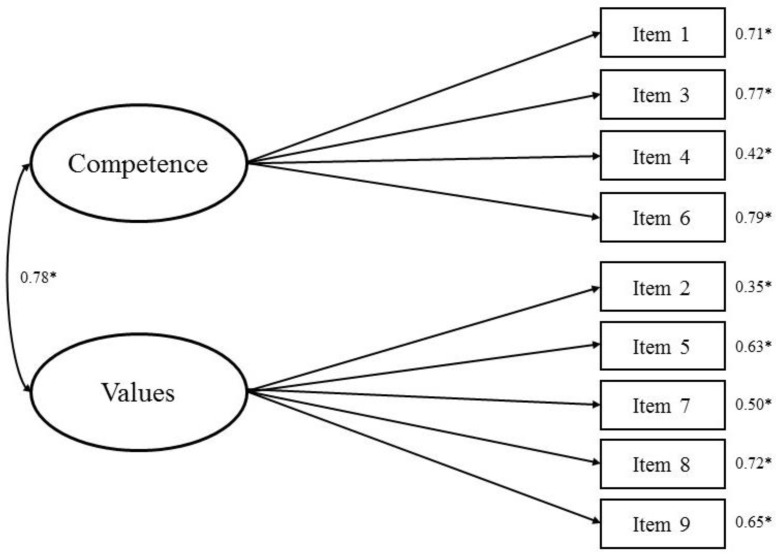
Confirmatory factor analysis results showing standardized estimates with errors for the Korean version of Health Care System Distrust Scale based on the two-factor model proposed by Shea et al. [22]. * *p* < 0.001.

**Figure 2:**
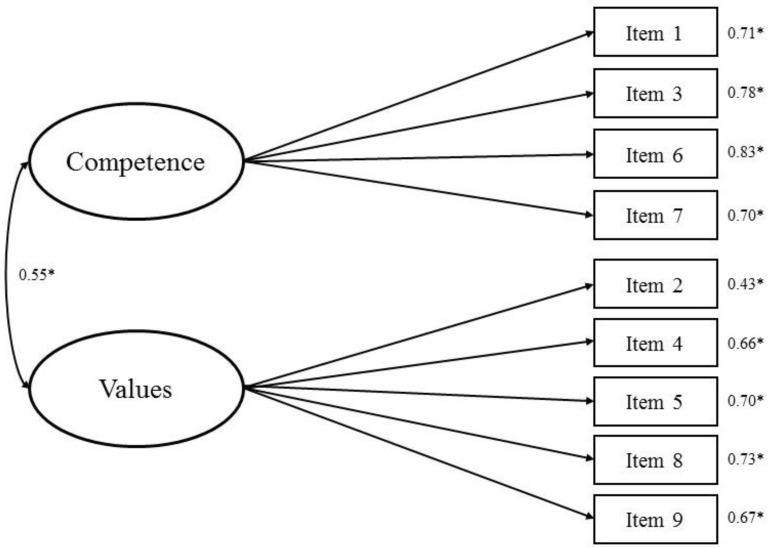
Confirmatory factor analysis results showing standardized estimates with errors for the Korean version of Health Care System Distrust Scale based on the modified two-factor model proposed by Yang et al. [10]. * *p* < 0.001.

The result of Barlett’s test of sphericity was significant (χ^2^(36) = 621.788, *p* < 0.001) and the Kaiser–Meyer–Olkin value was 0.84. Thus, the sample size was appropriate for factor analysis. As shown in Table 2, the fit for the HCSD-K scale using the two-factor model proposed by Shea et al. [22] was poor (χ^2^ = 165.32 (26), *p* < 0.001; χ^2^/*df* = 6.36; RMSEA = 0.165; SRMR = 0.107; CFI = 0.768; TLI = 0.679) (Figure 1). An excellent model fit for the HCSD-K scale was obtained using the modified two-factor model proposed by Yang et al. [10] (χ^2^ = 44.179 (26), *p* = 0.014; χ^2^/*df* = 1.58; RMSEA = 0.060; SRMR = 0.049; CFI = 0.970; TLI = 0.958). Therefore, this model should be preferred. The standardized regression coefficient weights of all items loading onto their respective factors were between 0.43 to 0.83, and all factor loadings of the items were statistically significant (Figure 2). Therefore, we adopted the modified two-factor model proposed by Yang et al. [10]. The HCSD-K scale is available in Supplementary S1 (Appendix A). 

We then examined the discriminant validity of the HCSD-K scale. The AVE values were 0.574 for competence distrust and 0.420 for values distrust. The squared correlation value between two factors was 0.229. Since the AVE values for each construct were greater than the shared variance estimate, there is no problem with the discriminant validity of the HCSD-K scale.

Convergent validity evidence is presented in Table 3. We correlated the composite scores for each subscale with the Perceived Discrimination in Health Care and Trust in Physician scale scores to assess the convergent validity of the HCSD-K scale. The results showed statistically significant positive correlations between scores for the Perceived Discrimination in Health Care scale and competence distrust (*r* = 0.523, *p* < 0.001) and values distrust (*r* = 0.581, *p* < 0.001) subscale scores, and statistically significant negative correlations between Trust in Physician scale scores and competence distrust (*r* = −0.662, *p* < 0.001) and values distrust (*r* = −0.570, *p* < 0.001) subscale scores. Thus, the convergent validity of the HCSD-K scale was supported.

### 3.4. Reliability 

The obtained Cronbach’s alpha coefficient signified the high reliability of the HCSD-K scale (Cronbach’s alpha = 0.83). The subscales (competence and values distrust) showed good internal consistency, with Cronbach’s alpha values of 0.84 and 0.77, respectively. The item-total correlation coefficients were >0.40 (range: 0.438–0.714). 

## 4. Discussion

This study described the process for translating and adapting the Revised HCSD scale into Korean and examined the scale’s psychometric properties. The findings indicate that the HCSD-K scale is an appropriate instrument to assess and measure the level of healthcare system distrust among KA women. We conducted a rigorous translation process using the committee-based translation method, cognitive interviews, and expert review to enhance the content validity of the instrument. 

We found that 76% of KA women had lived in the United States for more than 20 years, with a mean length of stay of 29 years, and most were naturalized citizens (80%). Approximately 85% participants had regular doctors, and 88% of participants had Korean doctors. The reason for the high percentage of patient-physician racial concordance could be due to low acculturation level. We have previously found that the low level of acculturation in this sample meaning that the participants were more affiliated with Korean culture and more comfortable with Korean language. [23] Consistent with this finding, previous studies showed that KAs prefer Korean doctors because of language proficiency, cultural sensitivity, understandability, and convenience. [25] 

The results from the CFA confirmed that the two-factor model proposed by Yang et al. [10] should be favored for the HCSD-K scale. The fit of the two-factor model for the HCSD-K scale proposed by Shea et al. [22] was poor. Thus, we performed the CFA based on the two-factor model proposed by Yang et al. [10] and the results indicated an excellent model fit. Therefore, we adopted the modified two-factor model created by Yang et al. [10] The CFA revealed that every item in the adopted HCSD-K scale contributed significantly to the scale’s domain, and that the construct validity of the scale was adequate. The discriminant validity of the competence distrust and values distrust subscales was supported, as the AVE values of each factor were larger than the shared variance estimate. Overall, this factor structure analyses implied that the modified two-factor model had the best model fit when applied with KA women. 

Our results supported the two hypotheses for convergent validity, based on moderate to strong correlations with other instruments. A positive association between perceived discrimination and the Revised HCSD scale was demonstrated in previous research. [47] Our study further examined the correlation between each HCSD-K subscale and perceived discrimination, with a slightly higher correlation between perceived discrimination and values distrust (*r* = 0.581, *p* < 0.001) than with competence distrust (*r* = 0.523, *p* < 0.001). Moreover, there was a significant negative association between scores for each subscale of the HCSD-K scale and scores for the Trust in Physician scale, confirming convergent validity. 

The results demonstrated the relatively high internal consistency of the HCSD-K scale (Cronbach’s alpha = 0.83), with alpha values of 0.84 and 0.77 for the competence and values distrust subscales, respectively. When compared to the Revised HCSD scale, the internal consistency was equivalent. Moreover, the item-total correlation coefficients were >0.40, indicating that the translated version is acceptable and that the items are less likely to be redundant. 

Although our study confirmed that the HCSD-K scale is a valid and reliable instrument to measure the level of healthcare system distrust among KA women, some limitations should be noted. The first limitation is the representativeness of the study findings. The convenience sampling from Korean churches in Chicago and the surrounding metropolitan areas and the cross-sectional design limit the generalizability of the study findings. However, churches are importance places for social and information exchanges in minority communities including KAs, thus it is difficult to assume that all church attendees are religious. [24,48] Nevertheless, our sample may overrepresented Christians and may have different trust levels than people with other religions, therefore the results should be interpreted with caution. For future studies, a random cluster sampling of Korean churches and other Korean organizations may be needed to increase generalizability. Second, the previous Revised HCSD scale was validated with a larger and younger sample with greater variability in socioeconomic status. Our study sample was limited to KA women who were aged between 50 and 74 years with limited sociodemographic variability. Future studies should include younger and mixed gender populations with greater variability in socioeconomic status. Lastly, the Revised HCSD scale was originally developed primarily with African Americans and whites. Further qualitative study or focus groups may be helpful to identify potential items that may be relevant to healthcare system distrust specific to the KA population. 

## 5. Conclusions

Trust in the healthcare system is a major contributor for racial disparities in health and health care and is known to affect healthcare utilization by minorities. Trust in the healthcare system has never been measured in KAs, as no valid and reliable measure was available. The study findings indicate that the HCSD-K scale is a reliable and valid instrument for researchers and healthcare providers to assess the level of trust in the healthcare system among KA women. Future research should replicate the study with a mixed gender KA population and other Asian populations.

## Figures and Tables

**Table 1:** Descriptive statistics of the participants (*N* = 196).

Characteristics	*n* (%)
Age (years)	
50–59	75 (38.2)
60–69	84 (42.9)
70–75	37 (18.9)
Residency in the United States (years)	
0–9	13 (6.6)
10–19	34 (17.4)
20–29	50 (25.5)
30–39	62 (31.6)
40 and above	37 (18.9)
Immigration status	
Immigrant (Naturalized)	156 (79.6)
Immigrant (Non-citizen)	34 (17.4)
Immigrant-Undocumented	3 (1.5)
Decline to answer	3 (1.5)
Marital status	
Married	147 (75.0)
Not married	49 (25.0)
Employment	
Part-time	27 (13.8)
Full-time	60 (30.6)
Not working	109 (55.6)
Education	
High school or less	80 (40.8)
College or higher	116 (59.2)
Income (US$)	
<10,000	18 (9.2)
10,000–24,999	28 (14.3)
25,000–39,999	44 (22.5)
40,000–54,999	22 (11.2)
55,000 or more	51 (26.0)
Do not know	15 (7.6)
Decline to answer	18 (9.2)
Regular doctor	
Yes	166 (84.7)
No	30 (15.3)
Physician race	
Korean	173 (88.3)
Non-Korean	23 (11.7)
Able to find Korean physician	
Yes	181 (92.4)
No	15 (7.6)
Able to receive care where wanted	
Yes	141 (71.9)
No	55 (28.1)

**Table 2:** Comparisons of model fit for the Korean version of the Health Care System Distrust scale.

Model	χ^2^	*df*	*p*-Value	RMSEA	SRMR	CFI	TLI
HCSD-K							
Shea two-factor model	165.32	26	<0.001	0.165	0.107	0.768	0.679
Yang two-factor model	44.18	26	0.014	0.060	0.049	0.970	0.958

Note. HCSD-K = Korean version of the Revised Health Care System Distrust; RMSEA = root mean square error of approximation; SRMR = standardized root mean residual; CFI = comparative fit index; TLI = Tucker–Lewis index.

**Table 3:** Convergent validity of the Health Care System Distrust scale.

	A	A1	A2	B	C
A. Health care system distrust	—				
A1. Competence	0.836 ***	—			
A2. Values	0.853 ***	0.427 ***	—		
B. Perceived discrimination in health care	0.655 ***	0.523 ***	0.581 ***	—	
C. Trust in physician	−0.728 ***	−0.662 ***	−0.570 ***	−0.671 ***	—

Note. *** *p* < 0.001

## Supplementary Materials

The following are available online at http://www.mdpi.com/1660-4601/15/9/1964/s1, Supplementary S1: Questionnaire.

## Appendix A

List of abbreviations: AVE: average variance extracted; CFA: confirmatory factor analysis; CFI: comparative fit index; HCSD: Revised Health Care System Distrust; HCSD-K: Revised Health Care System Distrust Scale—Korean; IRB: The Institutional Review Board; KA: Korean American; RMSEA: root mean square error of approximation; SRMR: standardized root mean residual; TLI: Tucker–Lewis index.
