# Supplementary material for: Translation and Validation of the Korean Version of the Revised Health Care System Distrust Scale (HCSD-K) in Korean American Women
Source: Int J Environ Res Public Health. 2018 Sep 8;15(9):1964. doi: 10.3390/ijerph15091964 (PMC6163931; doi:10.3390/ijerph15091964)
Supplement: Supplementary file 1 [file ijerph-15-01964-s001.pdf]

의료 시스템에 관한 불신

◈ **다음 질문들은 의료 시스템과 관련된 경험에 관한 것입니다. 의료 시스템은 건강관리와 관련된 모든 기관들, 즉 병원, 클리닉, 보험회사 등을 포함합니다. 각 문항들을 주의 깊게 읽고 해당하는 곳에 표시(√)해 주십시오.**

	전혀 동의 안함	동의 안함	동의도 부정도 안함	동의함	매우 동의함
1. 의료 시스템은 보다 나은 환자의 건강관리를 위해 최선을 다한다.	1	2	3	4	5
2. 의료 시스템은 그들의 실수를 은폐한다/감춘다.	1	2	3	4	5
3. 의료 시스템은 환자들에게 최고의 의료서비스를 제공한다.	1	2	3	4	5
4. 의료 시스템은 너무 많은 실수를 한다.	1	2	3	4	5
5. 의료 시스템은 환자가 필요로 하는 것 보다 돈 버는 것을 더 중요시 여긴다.	1	2	3	4	5
6. 의료 시스템은 훌륭한 의료서비스를 제공한다.	1	2	3	4	5
7. 환자의 인종/민족에 관계없이 의료시스템은 동일한 의료 서비스를 제공한다.	1	2	3	4	5
8. 의료시스템은 돈을 벌기 위해 거짓말을 한다.	1	2	3	4	5
9. 의료 시스템은 환자들에게 알리지 않고 의료 실험을 한다.	1	2	3	4	5
